# Deciphering molecular landscape of breast cancer progression and insights from functional genomics and therapeutic explorations followed by in vitro validation

**DOI:** 10.1038/s41598-024-80455-6

**Published:** 2024-11-20

**Authors:** Bushra Khan, Rowaid Qahwaji, Mashael S. Alfaifi, Tanwir Athar, Abdullah Khan, Mohammad Mobashir, Ibraheem Ashankyty, Khalid Imtiyaz, Areej Alahmadi, M. Moshahid A. Rizvi

**Affiliations:** 1 Department of Biosciences, Jamia Millia Islamia, New Delhi, India; 2 Department of Medical Laboratory Sciences, Faculty of Applied Medical Sciences, King Abdulaziz University, Jeddah, 22233 Saudi Arabia; 3 Hematology Research Unit, King Fahd Medical Research Center, King Abdulaziz University, Jeddah, Saudi Arabia; 4 Department of Epidemiology, Faculty of Public Health and Health Informatics, Umm Al-Qura University, Makkah, Saudi Arabia; 5 College of Dentistry and Pharmacy, Buraydah Private Colleges, Buraydah, 51418 Saudi Arabia; 6 Department of Mechanical Engineering, Faculty of Engineering, Jamia Millia Islamia, New Delhi, India; 7 Department of Biomedical Laboratory Science, Faculty of Natural Sciences, Norwegian University of Science and Technology (NTNU), Trondheim, 7491 Norway

**Keywords:** Ductal carcinoma in situ (DCIS), Invasive ductal carcinoma (IDC), Diagnostic markers, Fisetin, Gene expression, Breast cancer progression, Therapeutic exploration, Functional genomics, Cancer, Computational biology and bioinformatics

## Abstract

Breast cancer is caused by aberrant breast cells that proliferate and develop into tumors. Tumors have the potential to spread throughout the body and become lethal if ignored. Metastasis is the process by which invasive tumors move to neighboring lymph nodes or other organs. Metastasis can be lethal and perhaps fatal. The objective of our study was to elucidate the molecular mechanisms underlying the transition of Ductal Carcinoma In Situ (DCIS) to Invasive Ductal Carcinoma (IDC), with a particular focus on hub genes and potential therapeutic agents. Using Weighted Gene Co-expression Network Analysis (WGCNA), we built a comprehensive network combining clinical and phenotypic data from both DCIS and IDC. Modules within this network, correlated with specific phenotypic traits, were identified, and hub genes were identified as critical markers. Receiver Operating Characteristic (ROC) analysis assessed their potential as biomarkers, while survival curve analysis gauged their prognostic value. Furthermore, molecular docking predicted interactions with potential therapeutic agents. Ten hub genes—CDK1, KIF11, NUF2, ASPM, CDCA8, CENPF, DTL, EXO1, KIF2C, and ZWINT—emerged as pivotal fibroblast-specific genes potentially involved in the DCIS to IDC transition. These genes exhibited pronounced positive correlations with key pathways like the cell cycle and DNA repair, Molecular docking revealed Fisetin, an anti-inflammatory compound, effectively binding to both CDK1 and DTL underscoring their role in orchestrating cellular transformation. CDK1 and DTL were selected for molecular docking with CDK1 inhibitors, revealing effective binding of Fisetin, an anti-inflammatory compound, to both. Of the identified hub genes, DTL—an E3 ubiquitin ligase linked to the CRL4 complex—plays a central role in cancer progression, impacting tumor growth, invasion, and metastasis, as well as cell cycle regulation and epithelial-mesenchymal transition (EMT). CDK1, another hub gene, is pivotal in cell cycle progression and associated with various biological processes. In conclusion, our study offers insights into the complex mechanisms driving the transition from DCIS to IDC. It underscores the importance of hub genes and their potential interactions with therapeutic agents, particularly Fisetin. By shedding light on the interplay between CDK1 and DTL expression, our findings contribute to understanding the regulatory landscape of invasive ductal carcinoma and pave the way for future investigations and novel therapeutic avenues.

## Introduction

Ductal carcinoma in situ (DCIS) represents an early-stage breast cancer that is frequently identified through mammogram screenings, often devoid of overt symptoms. It is considered a pre-cancerous condition with the potential to progress into the more aggressive Invasive Ductal Carcinoma (IDC), the predominant form of breast cancer worldwide. IDC originates in the milk ducts and subsequently extends to neighboring breast tissues. IDC’s diagnosis frequently involves mammograms, ultrasounds, and biopsies. Treatment approaches encompass surgery, chemotherapy, radiation therapy, targeted therapy, and hormonal therapy, selected based on the stage and characteristics of the cancer (IARC 2020). However, breast cancer remains a pressing health concern in India, impacting both urban and rural populations and ranking as a prominent cause of cancer-related mortality among Indian women. Challenges within the healthcare system, including limited rural access, low awareness of early detection, cultural norms, and financial constraints, contribute to delayed diagnosis and treatment initiation^1–3^.

Histologically, IDC exhibits heterogeneous characteristics, with varying degrees of cellular differentiation. It can manifest as well-differentiated tumors, closely resembling normal breast ductal epithelium, or poorly differentiated tumors, featuring significant cellular and structural abnormalities^1,2^. The tumor microenvironment also plays a pivotal role, displaying a desmoplastic reaction characterized by collagen fiber accumulation and inflammatory cell infiltration^3^.

Molecularly, IDC’s complexity arises from the dysregulation of essential pathways such as PI3K/AKT/mTOR, ER/PR, HER2, TP53, EMT, and the cell cycle, leading to augmented cell growth, invasion, and metastasis (Arteaga and Engelman 2014). Targeting biomarkers associated with these pathways, including PIK3CA mutations, ER/PR status, HER2 overexpression, TP53 mutations, EMT markers, and cell cycle regulators, holds promise for therapeutic interventions (Nature 2012). However, addressing IDC’s challenges, including heterogeneity, treatment resistance, metastasis susceptibility, and side effects, remains demanding^4^.

In this context, systems biology, pharmacogenomics, and computational biology have emerged as invaluable tools for biomarker discovery and analysis^5,6^. These methodologies integrate diverse biological data, employ computational models, and facilitate personalized medicine, aiding in experimental design and validation^7^. High-throughput genomics technologies have revolutionized the field by allowing the simultaneous analysis of numerous genes or molecules^8,9^.

Weighted gene co-expression network analysis (WGCNA) is a particularly powerful approach in this regard. Leveraging high-throughput genomics data, WGCNA constructs gene co-expression networks, revealing patterns of gene expression and identifying functionally related gene groups termed modules. This analysis pinpoints hub genes with high connectivity scores that play central roles in coordinating other genes within a module, indicative of their involvement in significant biological processes^10–12^. In our study, we employed the weighted gene co-expression network analysis (WGCNA) approach to create an inclusive network integrating clinical and phenotypic data from cases of ductal carcinoma in situ (DCIS) and invasive ductal carcinoma (IDC) in breast cancer. Our primary objective was to identify network modules with correlations to specific phenotypic traits. We extensively investigated the associations between these modules and phenotypic traits, leading to the identification of hub genes as significant markers within the network. We evaluated their potential as biomarkers using Receiver Operating Characteristic (ROC) analysis and determined the prognostic value of these hub genes through survival curve analysis. Notably, a cluster of ten hub genes—CDK1, KIF11, NUF2, ASPM, CDCA8, CENPF, DTL, EXO1, KIF2C, and ZWINT—emerged as pivotal fibroblast-specific genes potentially involved in the transition from DCIS to IDC. These genes demonstrated substantial positive correlations with essential pathways like the cell cycle and DNA repair, highlighting their crucial roles in orchestrating cellular transformation. Of these, we focused on CDK1 and DTL for in-depth investigation, predicting their interactions with potential therapeutic agents, including CDK1 inhibitors, Doxorubicin, and Fisetin. Subsequent molecular docking analysis revealed an intriguing result: Fisetin, an anti-inflammatory compound, displayed a notable molecular binding affinity towards both CDK1 and DTL. This finding underscores Fisetin’s therapeutic potential, suggesting its capacity to interact molecularly with these core hub genes, potentially offering avenues for therapeutic interventions.

## Results

### Differential gene expression profiling of DCIS/IDC

**Fig. 1:**
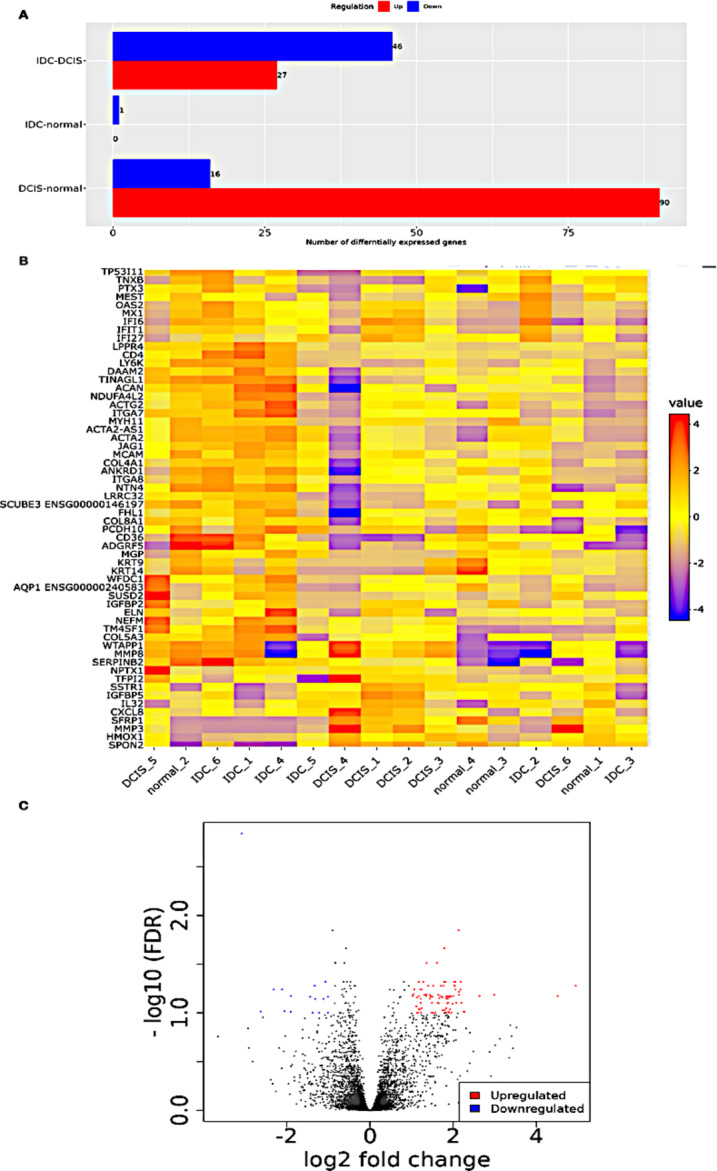
(**A**) Identification of Differentially Expressed Genes between DCIS/IDC and Normal Breast Samples (GSE228582). (**B**) Heatmap of Differentially Expressed Genes, indicating expression patterns in DCIS/IDC and Normal Breast Samples. (**C**) Volcano plots visually represent Differentially Expressed Genes (DEGs), with upregulated genes indicated by red nodes and downregulated genes shown as blue nodes. The scatter plot captures the presence of DEGs within the dataset. On the X-axis, the representation is of − log (adj.P.Val) (logarithm of adjusted p-value), while the Y-axis showcases the log2FC (logarithm of fold change). Genes with log2FC > 0.8 and − log(adj.P.Val) < 0.05, denoting upregulated genes, are depicted as magenta dots. Meanwhile, genes with log2FC < − 0.8 and − log (adj.P.Val) < 0.05, signifying downregulated genes, are represented by green dots.

The gene expression profiling yielded 936 differentially expressed genes (DEGs) in Ductal Carcinoma In Situ (DCIS) and Invasive Ductal Carcinoma (IDC) compared to the normal group. Among these DEGs, 590 genes were upregulated, and 346 genes were downregulated. The screening criteria for DEGs were set as log2 fold change ≥ 1.0 and False Discovery Rate (FDR) < 0.05. The volcano plot visually represents the distribution of the identified DEGs, highlighting the genes that exhibit significant changes in expression levels between the tumor samples (DCIS/IDC) and the normal samples. On the other hand, the heat map displays the results of two-way hierarchical clustering of the DEGs, illustrating how the gene expression patterns segregate the samples into different clusters based on their similarities or differences in expression profiles (Fig. 1A–C).

### Weighted co-expression network construction

**Fig. 2:**
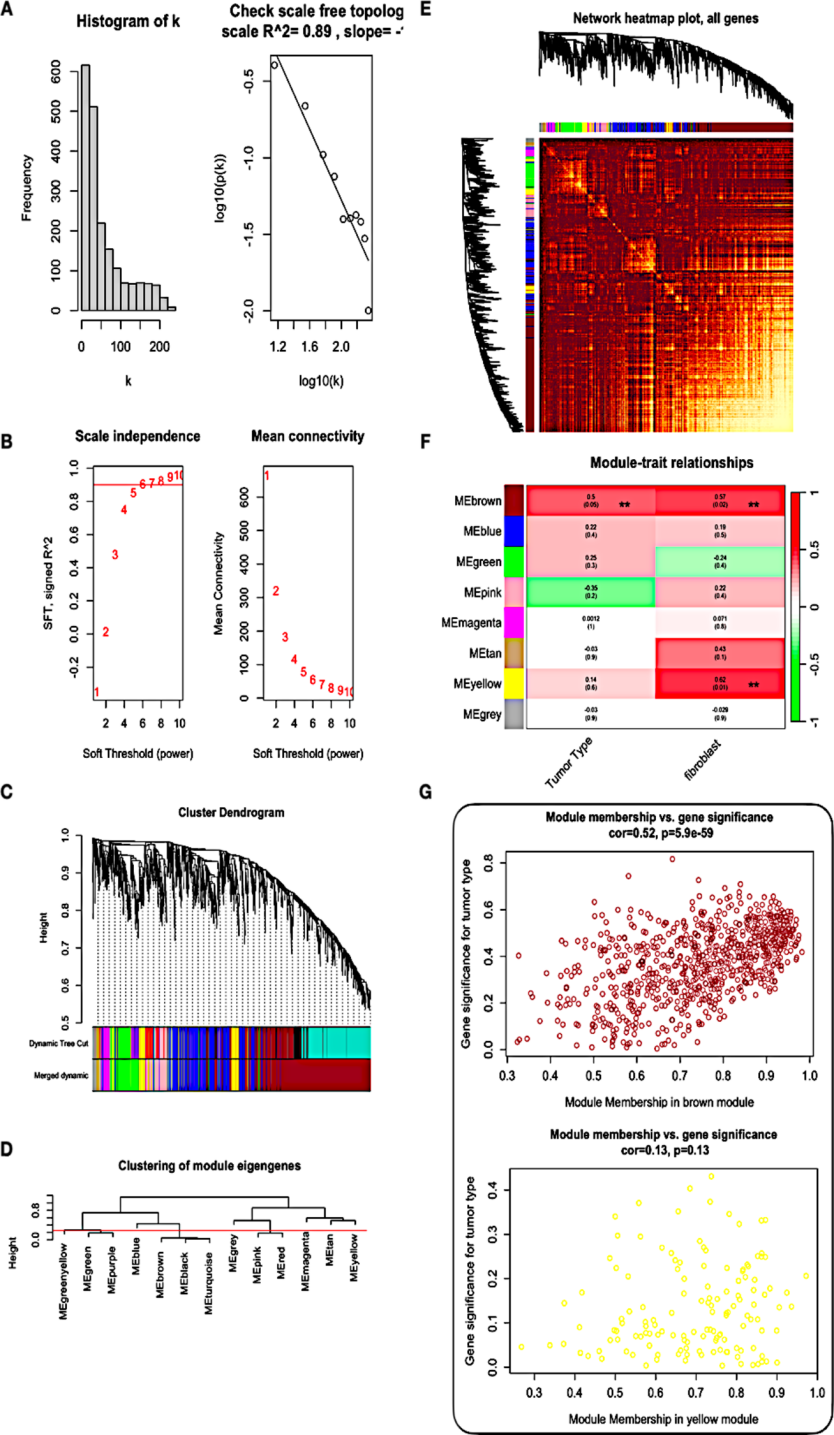
Construction of co-expression modules based on DCIS/IDC RNA-seq data from NCBI GEO database by WGCNA. (**A**,** B**) Analysis of network topology for various soft-threshold powers. Check scale-free topology; the adjacency matrix was defined using soft-thresholds with β = 6. Network topology analysis was performed under different soft threshold powers. The left panel shows the effect of soft threshold power on the scale-free topological fit index, evaluating the suitability of the network model. The right panel shows the influence of soft threshold power on the average connectivity fitting coefficient R2, providing insights into the interconnectedness of genes in the network. Mean connectivity is also shown as a function of the soft threshold parameter. (**C**) Clustering dendrograms of genes, with dissimilarity based on topological overlap, together with assigned module colors. The gene clustering tree (tree view) was constructed based on hierarchical clustering of adjacency differences. This tree-based clustering aids in identifying groups of genes with similar regulatory patterns. (**D**) Hierarchical clustering dendrogram of MEs and the threshold (red line) of modules need to be merged. (**E**) Heatmap depicting the topological overlap matrix (TOM) among genes based on co-expression modules. A redder background indicates a higher module correlation The co-expression network modules were examined using the Topological Overlap Matrix (TOM) plot. Light colors represent low gene overlap, while progressively darker red colors indicate higher overlap between genes, helping to identify gene clusters with similar expression patterns. (**F**) The Module-trait relationship heatmap illustrates the correlation between gene modules and different tumor types and fibroblast types. Rows correspond to modules, and columns correspond to specific tumor or fibroblast types. Each cell contains the correlation and p-value information, with red indicating positive correlation and blue indicating negative correlation. Modules significantly associated with tumor type and fibroblast type (|r| > 0.8 and p-value ≤ 0.05) are indicated by an asterisk. (**G**) The correlation between gene signatures for tumor type and module membership in the brown and yellow modules was examined, providing insights into the relationship between gene expression patterns and specific tumor types.

We applied the Weighted Gene Co-expression Network Analysis (WGCNA) algorithm to construct a co-expression network and identify modules within the gene expression data from the 16 samples. To achieve a scale-free topology with a coefficient of determination (R2) of 0.89 (Fig. 2A,B), the Pearson’s correlation matrix of the genes was converted into a strengthening adjacency matrix using a power parameter (β) of 6. The Dynamic Tree Cut algorithm was then employed to cluster the selected genes based on a topological overlap matrix (TOM)-based dissimilarity measure, leading to the division of the gene tree into eight distinct modules (Fig. 2C, D). Each module was represented by a unique color and contained a specific number of genes.

Furthermore, we have analyzed the interactions among these co-expression modules using Pearson’s correlation coefficient. Hierarchical clustering of module eigengenes, which summarizes the modules, revealed meta-modules represented by branches in the dendrogram (Fig. 2C). These meta-modules were grouped based on the correlation of eigengenes. Finally, to visualize the topological overlap between the genes within each module, we generated a heatmap plot (Fig. 2E,F). The heatmap showed different gene clusters within each module, with positive correlations represented in red and negative correlations in blue. The modularization and identification of gene clusters based on their co-expression patterns provide valuable insights into the regulatory relationships and functional associations within the gene expression data, potentially revealing key molecular pathways and biomarkers related to the studied conditions. While both the yellow and brown modules yielded significant p-values, it’s noteworthy that the yellow module exhibited an insignificant p-value in the module membership versus gene significance analysis (Fig. 2G). Hence, our focus was directed towards the brown module for the identification of hub genes (Tables 1, 2).

**Table 1:** PPI network cluster GO terms.

GO term	Description	Count in network	Strength	FDR
Cluster 1				
GO:0000226	Microtubule cytoskeleton organization	16 of 542	0.88	2.71E−07
GO:0000280	Nuclear division	15 of 323	1.07	4.84E−09
GO:0006139	Nucleobase-containing compound metabolic process	23 of 2722	0.33	0.0306
GO:0006259	DNA metabolic process	19 of 785	0.79	1.38E−07
GO:0006260	DNA replication	7 of 203	0.95	0.0037
Cluster 2				
GO:0006260	DNA replication	5 of 203	1.18	0.0345
GO:0006338	Chromatin remodeling	6 of 303	1.09	0.0202
GO:0006281	DNA repair	8 of 497	1.0	0.0056
GO:0006259	DNA metabolic process	11 of 785	0.94	0.00040
GO:0090304	Nucleic acid metabolic process	15 of 2203	0.62	0.0038

**Table 2:** Top 10 hub genes based on the top-rank order and their parameters.

Genes	GeneID	Description	Rank	Degree method		MCC		MNC	
				Name	Score	Name	Score	Name	Score
ZWINT	11,130	ZW10 interacting kinetochore protein	1	CDK1	46.0	CDK1	1.42014945313655E+17	CDK1	29.0
DTL	51,514	Denticleless E3 ubiquitin protein ligase homolog	2	KIF11	39.0	KIF11	1.42014945313655E+17	KIF11	29.0
EXO1	9156	Exonuclease 1	3	ASPM	38.0	NUF2	1.42014945309129E+17	CDCA8	29.0
CDK1	983	Cyclin dependent kinase 1	4	CDCA8	37.0	ASPM	1.42014944753711E+17	ASPM	28.0
CENPF	1063	Centromere protein F	5	NUF2	36.0	CDCA8	1.42014938599992E+17	NUF2	28.0
ASPM	259,266	Assembly factor for spindle microtubules	5	EXO1	36.0	CENPF	1.42014938599094E+17	EXO1	28.0
CDCA8	55,143	Cell division cycle associated 8	7	ZWINT	35.0	DTL	1.42013549895845E+17	CENPF	28.0
KIF2C	11,004	Kinesin family member 2 C	7	KIF2C	35.0	EXO1	1.4201354988891E+17	KIF2C	28.0
NUF2	83,540	NUF2 component of NDC80 kinetochore complex	7	CENPF	35.0	KIF2C	1.42012309837386E+17	ZWINT	27.0
KIF11	3832	Kinesin family member 11	10	DTL	34.0	MCM10	1.42009625907803E+17	DTL	27.0

### Functional enrichment analysis of genes in the brown module

**Fig. 3:**
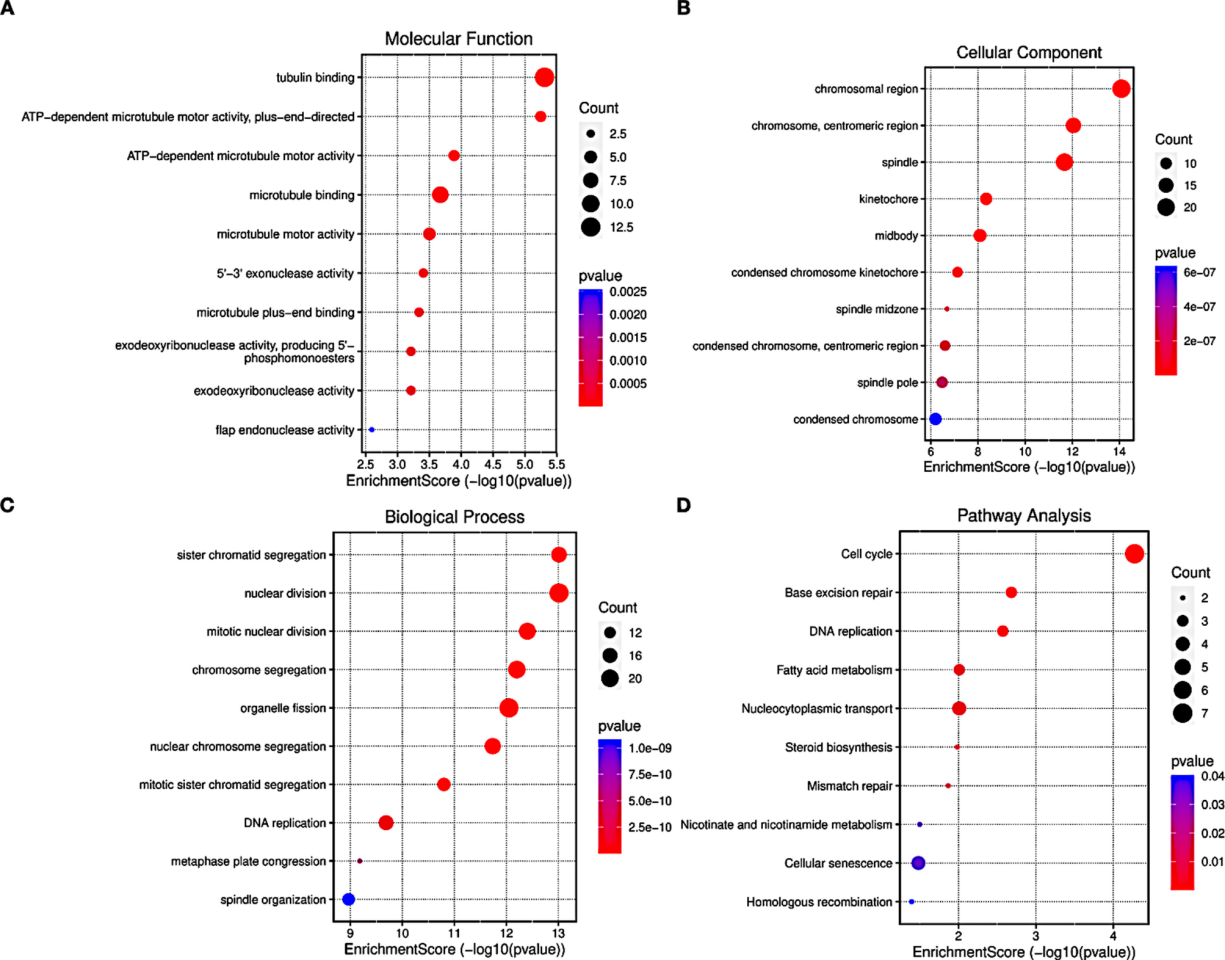
Functional analysis. (**A–C**) Gene Ontology (GO) enrichment analysis results of the genes in the brown module (https://www.bioinformatics.com.cn/en) In this module, there are genes from CC (Cellular Component), BP (Biological Process) and MF (Molecular Function) groups with a significantly increased expression. (**D**) Pathway enrichment analysis.

To assess the impacted functions associated with the genes clustered in the brown module, we conducted GO (Gene Ontology) and pathway analyses. In the present study, we performed enrichment analysis on the 150 key genes that displayed differential expression in DCIS/IDC tissues. The results of these analyses are presented in Fig. 3. To gain further insights into the differentially expressed genes (DEGs) and hub modules involved in biological processes (BP) terms and signaling pathways, we employed the clusterProfiler R package for GO and KEGG analyses (as depicted in Fig. 3A–D).The GO annotation revealed notable significance in several BP terms including sister chromatid segregation, nuclear division, mitotic nuclear division, chromosome segregation, organelle fission, nuclear chromosome segregation, mitotic sister chromatid segregation, DNA replication, metaphase plate congression, spindle organization, and more. Additionally, in the cellular component (CC) category, terms such as spindle, kinetochore, chromosomal region, spindle midzone, condensed chromosome, spindle pole, condensed chromosome centromeric region, and condensed chromosome kinetochore exhibited strong associations. The molecular function (MF) category was also linked to terms like tubulin binding, ATP-dependent microtubule motor activity, microtubule binding, microtubule motor activity, 5’–3’ exonuclease activity, microtubule plus end binding, exodeoxyribonuclease activity producing 5’ phosphomonoesters, exodeoxyribonuclease activity, flap endonuclease activity, among others, (Fig. 3). Moreover, the KEGG pathway analysis indicated that the common DEGs are interconnected with various pathways including the cell cycle, base excision repair, DNA replication, fatty acid metabolism, nucleocytoplasmic transport, steroid biosynthesis, mismatch repair, nicotinate and nicotinamide metabolism, cellular senescence, homologous recombination, and more.

### PPI network construction and hub gene identification

Then, we investigated the protein-protein interaction (PPI) network for the identified hub genes using the STRING database. The PPI network serves as a valuable tool to understand the interactions and functional relationships among these hub genes within the co-expression module (Table 1). Through the integration of the STRING database and the utilization of Cytoscape software, we successfully mapped the 150 differentially expressed genes (DEGs) from the module into the PPI network. This network presentation offered an effective means to visualize protein-protein interactions and unveil potential molecular mechanisms. The PPI network, composed of DEGs, revealed two functionally enriched clusters ranked by scores. To gain insights into their roles, we conducted a Gene Ontology (GO) and pathway enrichment analysis for each cluster (as depicted in Fig. 3A–D). Cluster 1, with 77 nodes and 458 edges, was implicated in processes such as Microtubule cytoskeleton organization, Nuclear division, Nucleobase-containing compound metabolic processes, among others. On the other hand, Cluster 2, consisting of 31 nodes and 13 edges, appeared to be involved in functions related to DNA replication, Chromatin remodeling, DNA repair, DNA metabolic processes, and Nucleic acid metabolic processes, all with significant statistical significance (Table 2).

**Fig. 4:**
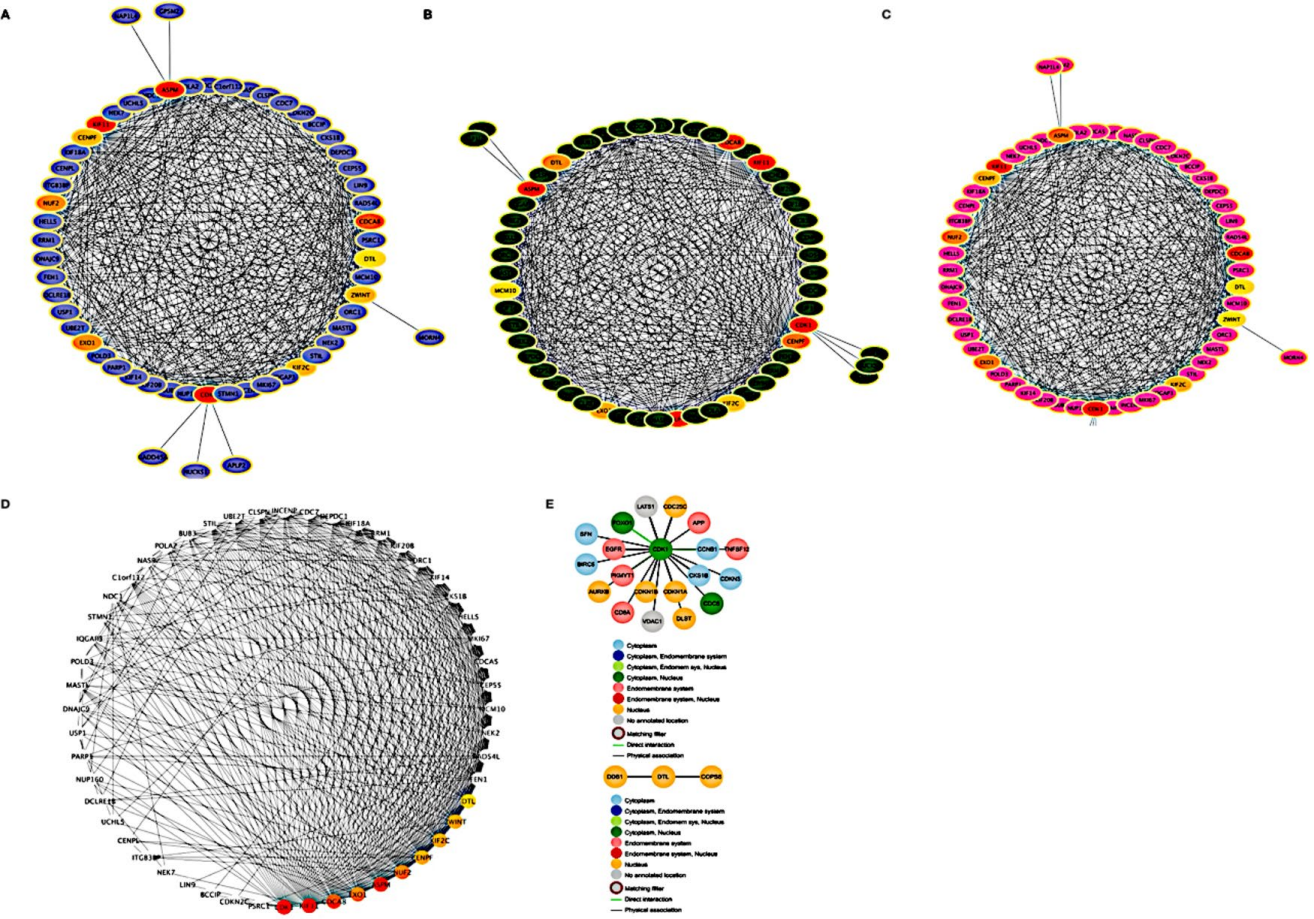
Hub genes network visualization. (**A–C**) Degree, MNC, and MCC of hub genes. Three methods (Degree, MNC, and MCC) were used to identify the hub genes. (**D**) Network of all the genes in brown module. MCC methods showed different hub genes. Between them, a comparison method was deployed to identify common hub genes, and the ten common genes selected as hub genes were as follows: ZWINT, DTL, EXO1, KIF11, CENPF, ASPM, KIF2C, NUF2, CDCA8 and CDK1. The networks shown here were drawn by using cytoscape^13^. (**E**) Protein–protein interactions of CDK1 and DTL with different proteins.

To visualize the PPI network, we utilized Cytoscape software. To identify hub genes, we utilized three different methods: Degree, MNC (Maximum Neighborhood Component), and MCC (Maximal Clique Centrality). The results from these three methods (Degree, MNC, and MCC) revealed distinct hub genes. In order to identify common hub genes, we conducted a comparison across these methods. As a result, we identified ten genes that consistently appeared as hub genes across all three methods: ZWINT, DTL, EXO1, KIF11, CENPF, ASPM, KIF2C, NUF2, CDCA8, and CDK1 (Fig. 4).

Through the graphical representation generated by Cytoscape (as depicted in Fig. 4A–E), the results unveiled several hub genes situated within this module. This set of hub genes includes CDK1, KIF11, NUF2, ASPM, CDCA8, CENPF, DTL, EXO1, KIF2C, and ZWINT. Notably, within this hub gene network, CDK1 and DTL exhibited the highest node values, underscoring their central roles in mediating interactions within the network. Due to their pronounced prominence and significance within the PPI network, CDK1 and DTL were singled out as the core hub genes warranting further investigation. It’s highly likely that these genes play critical regulatory roles in the molecular pathways and biological processes associated with the progression and development of the conditions under study. As such, they hold great potential as subjects for further research and as prospective targets for therapeutic interventions.

### Identification of transcription factors (TFs) within the key module

**Fig. 5:**
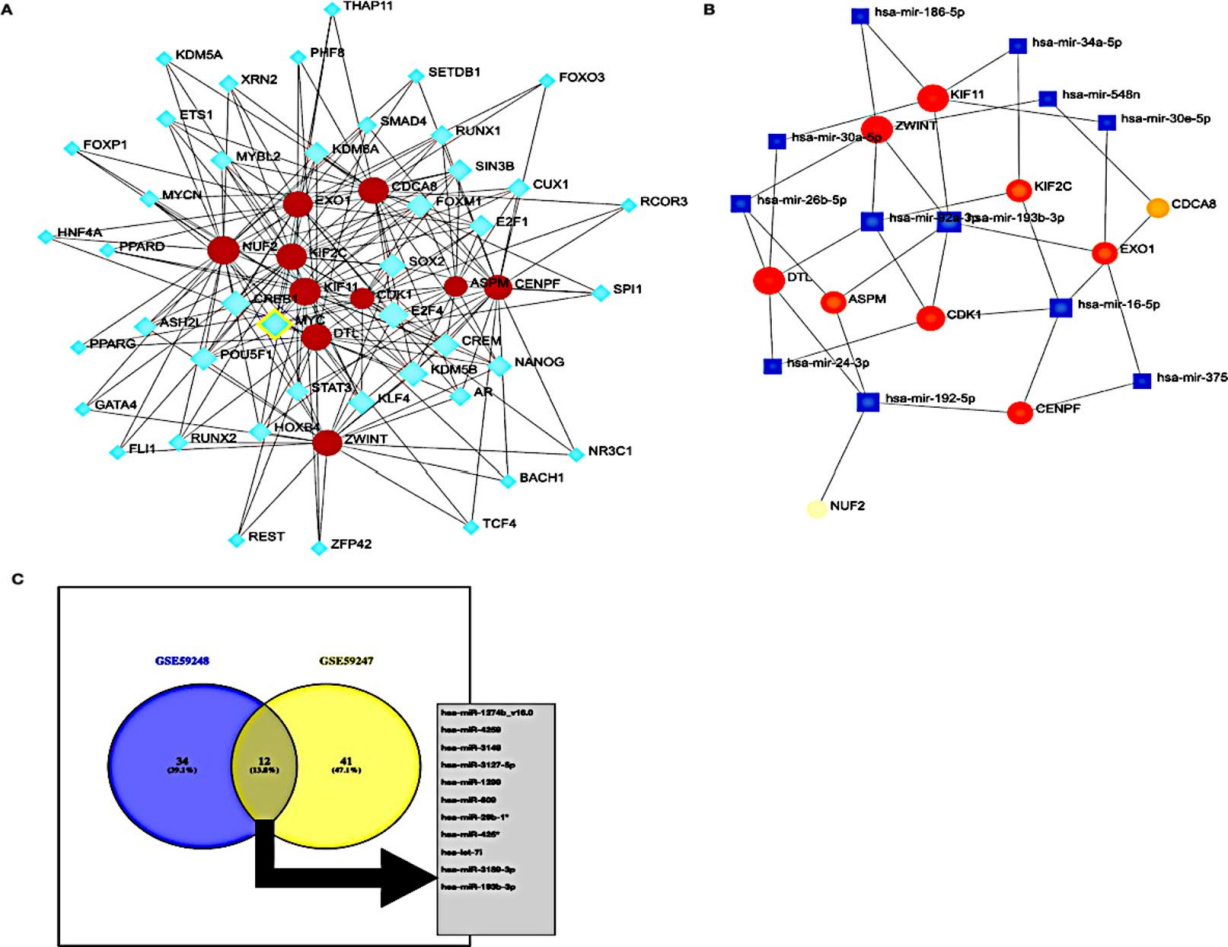
Networks of TFs and the miRNAs. (**A**) Transcription factors (TFs) regulatory network for the genes in the brown module. Blue diamonds represent the transcription factors, and red nodes represent the genes (generated by NetworkAnalyst). (**B**) MicroRNA-target regulatory network for the brown module. Blue rectangles represent the microRNAs, and red nodes represent the genes. The node size indicates the degree in the network (generated by NetworkAnalyst). (**C**) Venn diagram (source: venny2.0) illustrating the overlap of microRNAs identified in two datasets: GSE59248 (blue circle) and GSE59247 (yellow circle). The blue circle contains 34 unique microRNAs (39.1%), while the yellow circle contains 41 unique microRNAs (41.1%). The intersection of the two circles represents 12 shared microRNAs (13.8%) between the datasets.

To determine whether TFs may be responsible for the observed altered gene expression in the brown module, we inspected three data sources available in Network Analyst platform (https://www.networkanalyst.ca/NetworkAnalyst/home.xhtml)^14^. Three data sources, namely, ENCODE, JASPAR, or ChEA. Our comprehensive analysis unveiled a total of 43 TFs prominently associated with the brown module (as depicted in Fig. 5A). These TFs encompassed a diverse array of regulatory proteins with distinct functions. Among the identified TFs, noteworthy mentions include E2F4 (E2F Transcription Factor 4), MYC (MYC Proto-Oncogene), KDM5B (Lysine Demethylase 5B), CREB1 (CAMP Response Element Binding Protein 1), SOX2 (SRY-Box Transcription Factor 2), KLF4 (Krüppel-Like Factor 4), POU5F1 (POU Domain Class 5 Transcription Factor 1), FOXM1 (Forkhead Box M1), CREM (CAMP Responsive Element Modulator), KDM6A (Lysine Demethylase 6 A), NANOG (Nanog Homeobox), E2F1 (E2F Transcription Factor 1), SIN3B (SIN3 Transcription Regulator Family Member B), AR (Androgen Receptor), STAT3 (Signal Transducer and Activator of Transcription 3), RUNX1 (RUNX Family Transcription Factor 1), CUX1 (Cut Like Homeobox 1), MYBL2 (MYB Proto-Oncogene Like 2), ASH2L (ASH2 Like Histone Lysine Methyltransferase Complex Subunit), HOXB4 (Homeobox B4), SMAD4 (SMAD Family Member 4), and MYCN (MYCN Proto-Oncogene), to name a few.

These identified TFs exhibited varying degrees of connectivity, each potentially influencing a distinct set of target genes. For instance, E2F4 had 9 target genes identified, MYC demonstrated association with 9 target genes, and BHLH (Transcription Factor) was linked to 9 target genes as well. This trend continued with KDM5B, CREB1, SOX2, KLF4, POU5F1, FOXM1, CREM, KDM6A, NANOG, E2F1, SIN3B, AR, STAT3, RUNX1, CUX1, MYBL2, ASH2L, HOXB4, SMAD4, MYCN, SPI1, ETS1, XRN2, RUNX2, PPARG, NR3C1, RCOR3, PPARD, FOXO3, SETDB1, TCF4, HNF4A, BACH1, PHF8, THAP11, FOXP1, REST, and ZFP42, each linked with varying numbers of target genes. These TFs played a pivotal role in various biological pathways, including Cellular Senescence, Transcriptional deregulation in Cancer, Acute Myeloid Leukemia, Pathways in Cancer, Cell Cycle, and HTLV-I Infection, underscoring their potential involvement in key regulatory processes associated with cancer and cellular dynamics.

### miRNA-target regulatory network

By using the TargetScan, miRTarBase, miRecords databases, in Network Analyst we were able to identify the 22 most common miRNAs responsible for regulating the target genes in the brown module (Fig. 5B, C). The greatest number of genes were regulated miR-193b-3p with (Betweenness = 3046.165). Thus, these results indicated that miR-193-3p may serve a role in DCIS/ progression.

To analyze differentially expressed genes (DEGs) in the GSE59248 and GSE59247 datasets, we utilized the GEO2R tool, an interactive platform for gene expression analysis. We accessed both datasets from the Gene Expression Omnibus (GEO) with a focus on comparing ductal carcinoma in situ (DCIS) and invasive ductal carcinoma (IDC) samples. Using GEO2R, we defined sample groups based on experimental conditions and performed differential expression analysis. We selected appropriate statistical parameters, including the false discovery rate (FDR) for multiple testing correction and log2 transformation of the data. This analysis generated a list of DEGs ranked by significance, allowing us to identify genes with adjusted p-values below a threshold of 0.05.

To further investigate the relationships among the identified DEGs from both datasets, we created a Venn diagram, which revealed common miRNAs associated with breast cancer progression. The overlapping miRNAs included hsa-miR-1274b, hsa-miR-4259, hsa-miR-3148, hsa-miR-3127-5p, hsa-miR-1299, hsa-miR-609, hsa-miR-29b-1*, hsa-miR-425*, hsa-let-7i, hsa-miR-3189-3p, and notably, hsa-miR-193b. This highlighted the expression of hsa-miR-193b-3p^15^ in breast cancer datasets (Fig. 4F).

miR-193b-3p has been recognized as an anti-metastatic microRNA, with expression levels significantly reduced in metastatic breast cancer cells compared to non-metastatic counterparts, suggesting its role as a metastasis suppressor^16^. It targets critical genes involved in key signaling pathways such as Wnt and TGF-β, which influence cancer cell migration and invasion. Specifically, miR-193b-3p inhibits dimethylarginine dimethylaminohydrolase 1 (DDAH1), a regulator of angiogenesis and vasculogenic mimicry in aggressive breast cancer cells. Additionally, aberrant expression patterns of miR-193b-3p correlate with clinicopathological features like tumor differentiation and TNM staging, indicating its potential as a prognostic biomarker in breast cancer. Restoring miR-193b-3p levels in metastatic cells has been proposed as a therapeutic strategy to inhibit invasive behavior and improve treatment outcomes^17,18^.

### Identification and validation of key genes with poor prognosis for DCIS/IDC transition

**Fig. 6:**
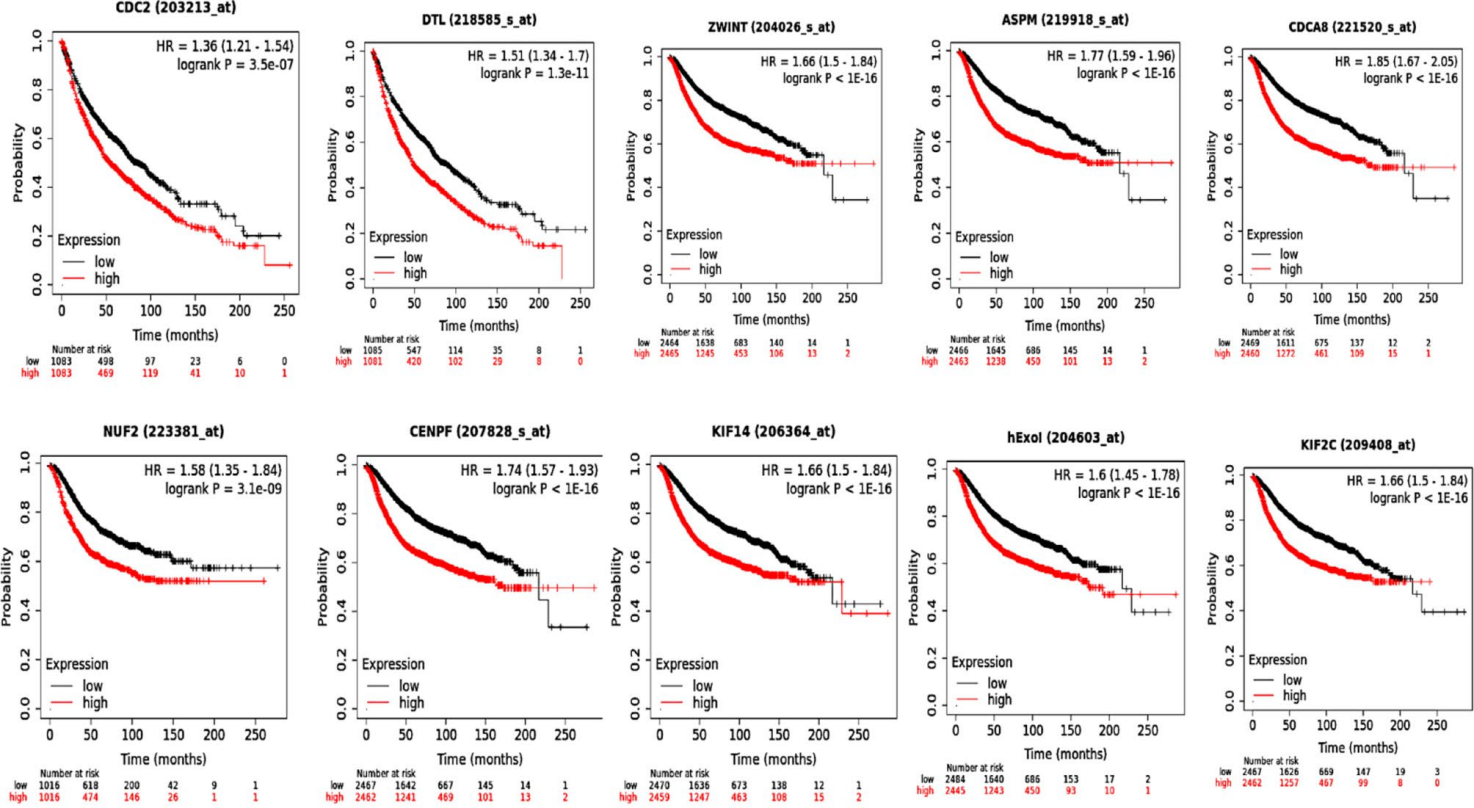
The overall survival of 10 hub genes in the Cancer Genome Atlas (TCGA) database (source: KM plotter (https://kmplot.com/analysis/)).

Based on our findings above, a set of ten genes comprising CDK1, KIF11, NUF2, ASPM, CDCA8, CENPF, DTL, EXO1, KIF2C, and ZWINT have been identified as potential hub genes. To further validate and refine these findings, survival analysis using Kaplan–Meier plotting was performed on patients with DCIS/IDC, revealing a significant correlation between higher expression of these candidate hub genes and poorer patient survival (Fig. 6).

**Fig. 7:**
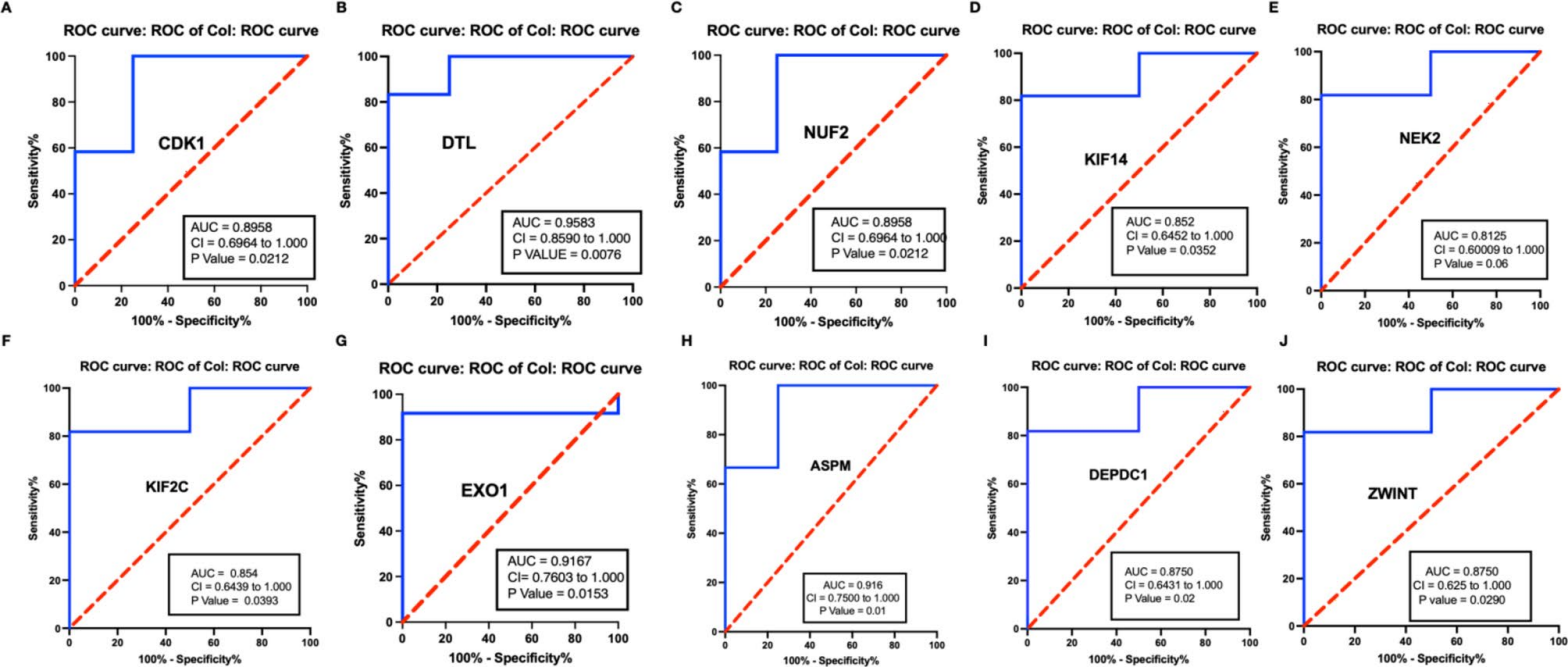
ROC curves for identified potential hub genes, respectively. These curves illustrate the sensitivity and specificity of DTL and CDK1 as potential biomarkers in distinguishing between different conditions or disease states. The ROC curves help assess the diagnostic accuracy of each gene’s expression levels in predicting specific outcomes.

To bolster the robustness of our findings and to assess the clinical applicability of the candidate hub genes as prognostic biomarkers, an additional layer of scrutiny was employed. To further test the value of the candidate hub genes as prognostic biomarkers of IDC, ROC curves were plotted and the AUC (95% CIs) were calculated (Fig. 7A–J). The results indicated that ten genes exhibited a high predictive accuracy for the development of IDC after DCIS, including CDK1 (cyclin dependent kinase 1), KIF11 (kinesin family member 11), NUF2 (NUF2 component of NDC80 kinetochore complex), ASPM (assembly factor for spindle microtubules), CDCA8 (cell division cycle associated 8), CENPF (centromere protein F), DTL (denticle-less E3 ubiquitin protein ligase homolog), EXO1(exonuclease 1), KIF2C (kinesin family member 2 C), and ZWINT (ZW10 interacting kinetochore protein). Hence, ten genes were regarded as the real hub genes in the WGCNA.

**Fig. 8:**
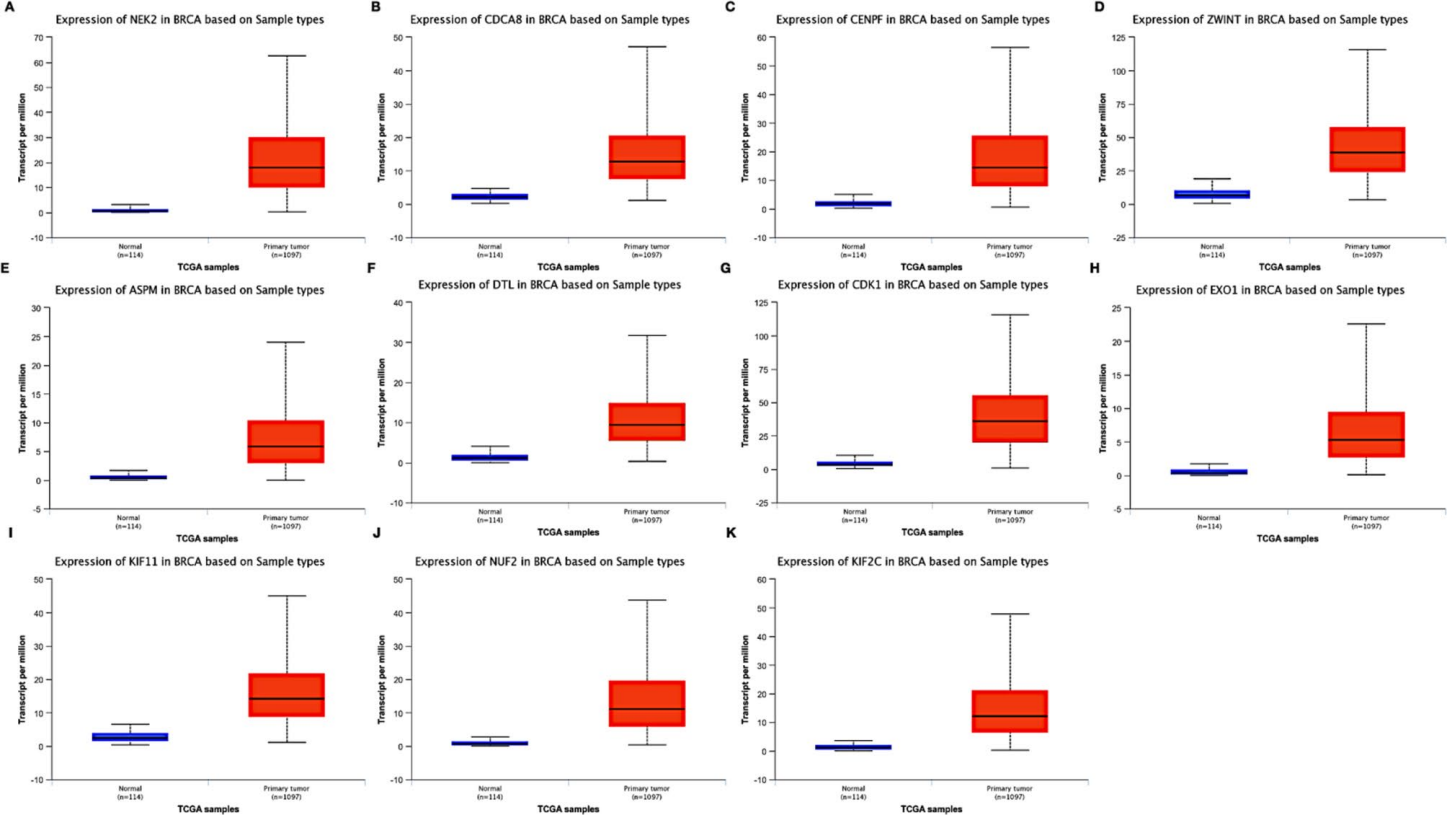
The validation of the mRNA expression level of hub genes. (**A–K**). The mRNA expression level of Ten hub genes in the Cancer Genome Atlas (TCGA) (Source: UALCAN).

To reinforce the validity of the genes identified through the afore-mentioned methods, the expression levels of these specific genes were examined using UALCAN (https://ualcan.path.uab.edu/). This analysis demonstrated elevated expression of CDK1, KIF11, NUF2, ASPM, CDCA8, CENPF, DTL, EXO1, KIF2C, and ZWINT in DCIS/IDC samples compared to normal breast tissue (Fig. 8A–K).

### Gene correlation analysis in cBioPortal

To validate co-expressed hub genes and identify potential oncogenes in breast cancer, we utilized the cBioPortal online platform (https://www.cbioportal.org/) to analyze the co-expression of CDK1 and DTL^19,20^. This analysis was conducted using data from the TCGA dataset, which encompasses 13,146 samples across 30 studies within the Breast Carcinoma (TCGA provisional) sample set. We applied a Bonferroni adjustment for multiple testing, setting the cutoff for statistical significance at a p-value of < 0.01 (Table 3).

**Table 3:** The significant gene co-expression pairs among the hub genes.

A	B	Neither	A not B	B not A	Both	Log2 odds ratio	p-value	q-value	Tendency
EXO1	DTL	5331	201	120	424	> 3	< 0.001	< 0.001	Co-occurrence
CENPF	DTL	5367	165	34	510	> 3	< 0.001	< 0.001	Co-occurrence
ASPM	DTL	5262	270	147	397	> 3	< 0.001	< 0.001	Co-occurrence
NUF2	DTL	5291	241	196	348	> 3	< 0.001	< 0.001	Co-occurrence
CDK1	ZWINT	5866	112	44	54	> 3	< 0.001	< 0.001	Co-occurrence
CDK1	CENPF	5286	115	624	51	1.909	< 0.001	< 0.001	Co-occurrence
CDK1	KIF11	5850	150	60	16	> 3	< 0.001	< 0.001	Co-occurrence
CDK1	DTL	5408	124	502	42	1.867	< 0.001	< 0.001	Co-occurrence
KIF2C	DTL	5458	74	515	29	2.054	< 0.001	< 0.001	Co-occurrence
CDK1	ASPM	5284	125	626	41	1.469	< 0.001	< 0.001	Co-occurrence
CDK1	KIF2C	5821	152	89	14	2.591	< 0.001	< 0.001	Co-occurrence
CDK1	CDCA8	5831	154	79	12	2.524	< 0.001	< 0.001	Co-occurrence
CDCA8	DTL	5463	69	522	22	1.738	< 0.001	< 0.001	Co-occurrence
KIF11	DTL	5472	60	528	16	1.467	< 0.001	0.001	Co-occurrence
ZWINT	DTL	5452	80	526	18	1.222	0.003	0.003	Co-occurrence

**Fig. 9:**
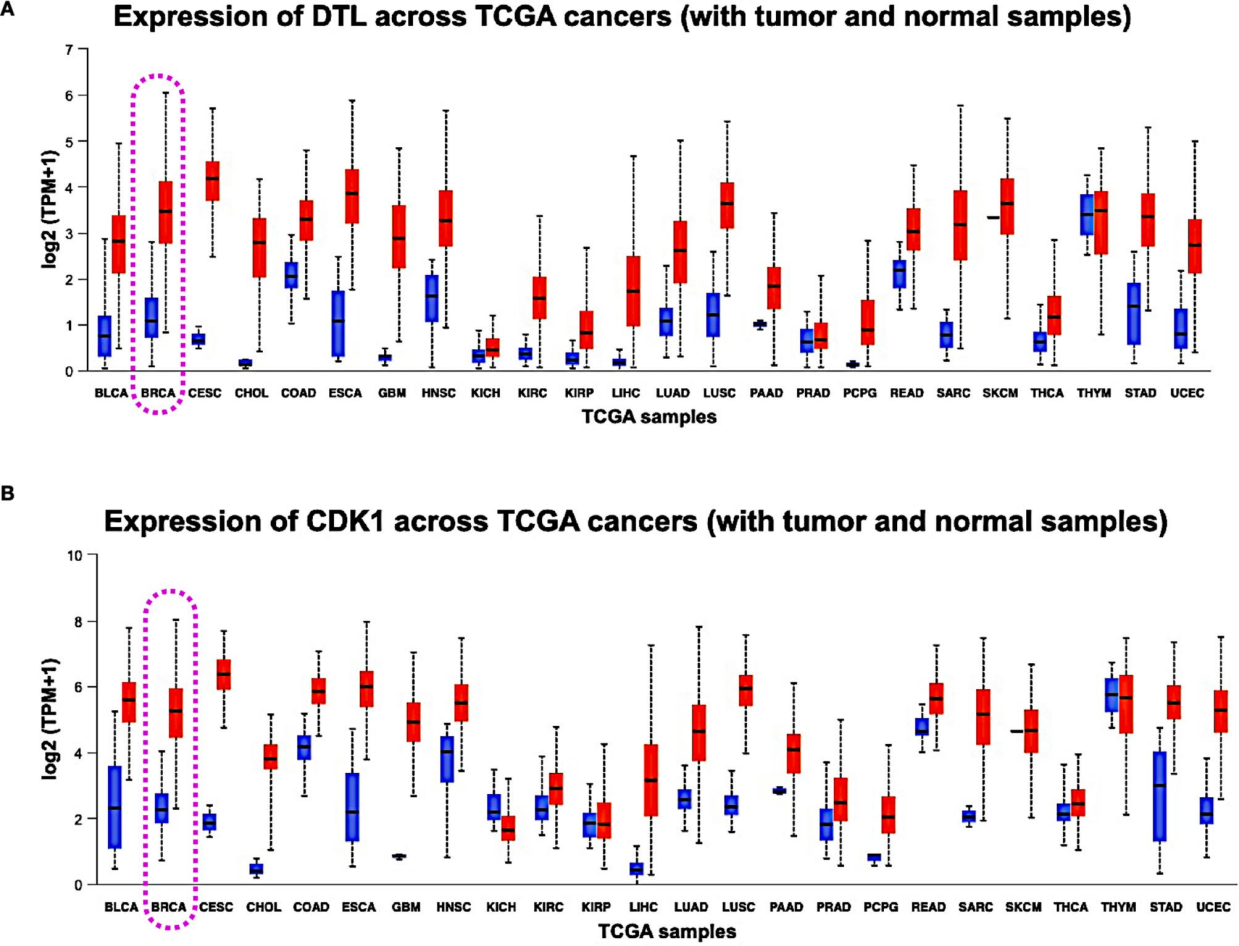
In different TCGA studies, the expression levels of (**A**) DTL and (**B**) CDK1 were investigated. The expression for breast cancer is highlighted with dotted box.

### Gene expression of CDK1 and DTL across different cancer types

By utilizing the UALCAN server, an extensive investigation was conducted across a spectrum of TCGA studies, covering a wide range of cancer types such as LAML, ACC, BRCA, CHOL, LCML, COAD, GBM, HNSC, KICH, KIRC, LUSC, and more. This comprehensive inquiry aimed to unravel the intricate expression patterns of CDK1 and DTL across these diverse cancer studies. Particularly noteworthy was the consistent and striking finding within the BRCA study: the expression levels of both CDK1 and DTL were markedly elevated in tumor samples compared to other conditions (Fig. 9A and B).

### Docking analysis

The molecular docking verification of active compounds and key proteins was conducted using AutoDock Vina, a prominent open-source software designed for molecular docking that enhances binding mode prediction accuracy compared to its predecessor, AutoDock 4 (https://vina.scripps.edu/). Active compounds were sourced from the PubChem database. Following this, AutoDock Vina facilitated the docking of potential small-molecule active compounds with primary target proteins associated with ductal carcinoma in situ (DCIS) and invasive ductal carcinoma (IDC). The efficacy of these compounds was assessed based on their binding free energy, with results visualized using PyMOL, a molecular visualization tool.

**Fig. 10:**
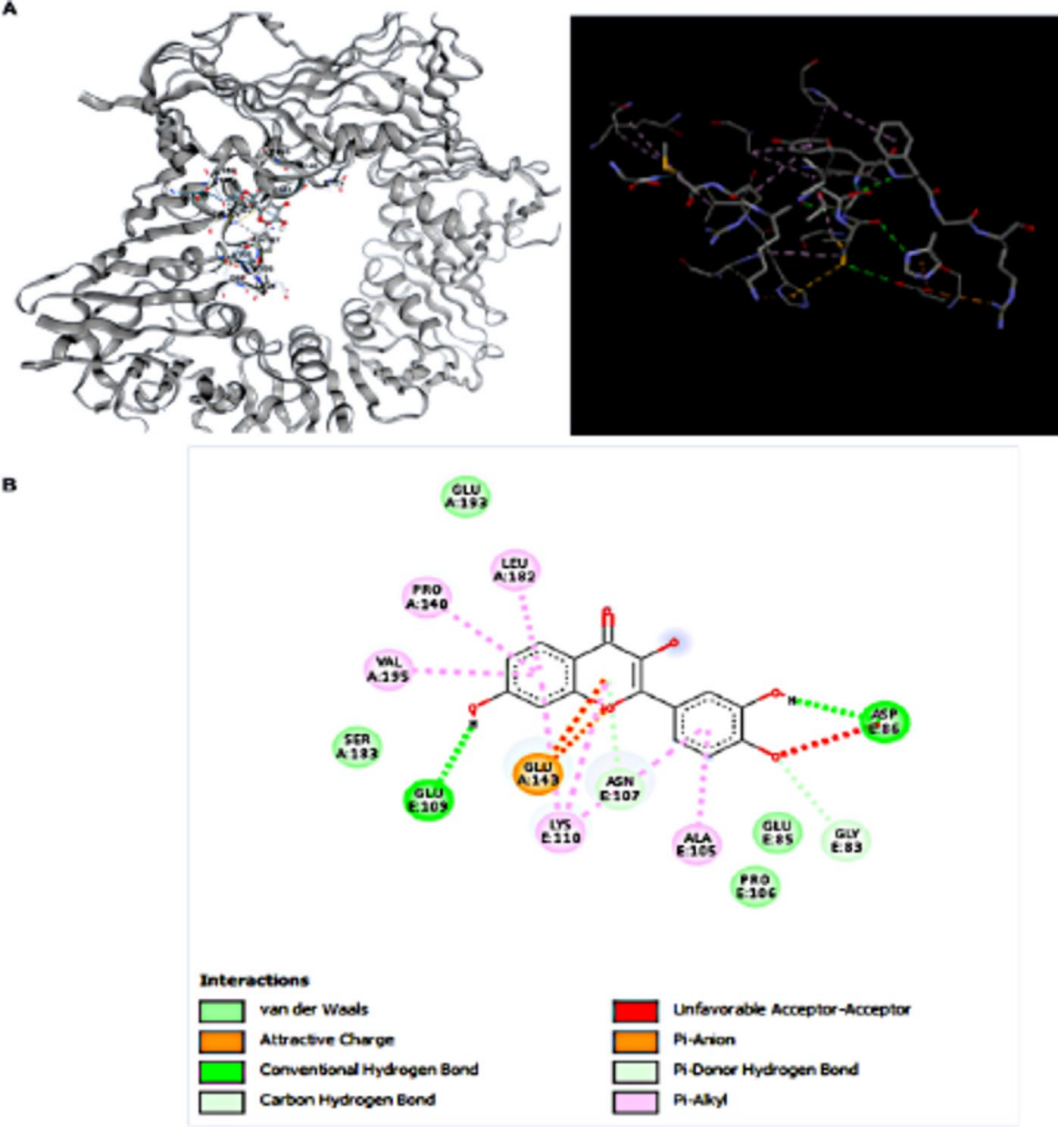
Molecular docking analysis of Fisetin and DTL protein (**A**) 3D Diagram: This representation illustrates the spatial orientation of Fisetin when bound to the DTL protein. The binding site is highlighted, showcasing the interaction between the ligand and the protein structure. (**B**) 2D Diagram: This schematic representation illustrates the specific binding interactions of Fisetin with the DTL protein, highlighting both hydrogen bonds and hydrophobic contacts. Notably, Fisetin forms strong hydrogen bonds with the amino acid residues ASP86 and GLU109, while exhibiting weaker hydrogen bonds with ASN107. Furthermore, hydrophobic interactions are observed with residues ALA105, ASN107, LYS110, PRO140, GLU143, and VAL195. This detailed interaction profile underscores the complexity of Fisetin’s binding mechanism with DTL.

In this study, the binding stability of CDK1 inhibitors to CDK1 and DTL were assessed using molecular docking techniques. Generally speaking, a binding energy less than − 5.0 kcal/mol indicates an excellent binding and less than − 7.0 kcal/mol signify strong binding interactions^21^. The molecular docking results were shown in (Table 5), from which it could be observed that there were strong binding interactions between CDK1 inhibitors and DTL gene, with the strongest direct in Fisetin-DTL binding (Tables 4, 5). The 3D schematic of CDK1 Inhibitors binding with DTL is displayed in (Fig. 10A, B). The results, presented in Table 4, demonstrated robust binding interactions between CDK1 inhibitors and the DTL gene, particularly highlighting the strongest interactions observed with DTL-Doxorubicin, DTL-Alsterpaullone, and DTL-Fisetin. Given the availability of Fisetin and its supporting literature, it was selected for further in vitro analysis^22^.

**Table 4:** Drugs used for molecular docking studies.

Drugbank ID	Name	Actions	Drug group
DB07795	Fisetin	Binder	Experimental
DB00997	Doxurubicin	Inhibitor	Approved, Investigational
DB03496	Alsterpaullone	Inhibitor	Experimental
DB03496	Alvocidib	–	Experimental
DB02950	Hymenialdisine	–	Experimental
DB02052	Indirubin-3’-monoxime		Experimental
DB02116	Olomoucine		Experimental
DB08142	AT-7519	–	Investigational
DB02010	Fostamatinib	Inhibitor	Approved, Investigational
DB16652	Avotaciclib	Inhibitor	Investigational

**Table 5:** Predicted binding sites and the scores details for the respective proteins and the drugs combinations.

Compound	Binding energy (Kcal/mol)	Cavity size	Centre (x, y,z)	Size (x × y × z)	Hydrogen bonding, Hydrophilic interactions with following amino acids	Compound	Binding energy (Kcal/mole)	Cavity size	Centre (x, y,z)	Size (x × y × z)	Hydrogen bonding, Hydrophilic interactions with following amino acids
DTL-Fisetin	− 7.4	625	48, 35, − 21	21 × 21 × 21	PRO140 SER141 GLY142 GLU143 ARG146 ARG149 ASP150 LYS181 LEU182 SER183 GLN184 THR185 SER186 ASN187 VAL188 GLU192 GLU193 ALA194 VAL195 SER223	CDK1-FISETIN	− 8.4	15,057	(− 13, − 4, − 30)	35 × 35 × 31	ILE10 GLY11 GLU12 GLY13 VAL18 ALA31 LYS33 PHE82 LEU83 SER84 ASP86 LEU87 LYS88 LYS89 LEU91 ASP92 ARG127 ASP128 PRO131 GLN132 ASN133 LEU135 ALA145 ASP146 ARG151 ALA152 PHE153 GLY154 THR161 HIS162 VAL164 VAL165 THR166 TRP168 TYR169 ARG170 GLU196 LYS200 PRO202 HIS205 GLU209
DTL-DOXURUBICIN	− 7.9	502	36, − 8, 15	24 × 24 × 24	LEU 126, GLU 124, GLN 38, LEU 47, SER 42, HIS 44, SER 39, MET 40	CDK1-DOXURUBICIN	− 9.1	2101	0, 0, − 34	24 × 35 × 24	ARG44 VAL48 GLN49 ILE10 GLY11 GLU12 GLY13 THR14 VAL18 ALA31 MET32 LYS33 GLU38 VAL64 PHE80 GLU81 PHE82 LEU83 SER84 LYS89 ARG127 LYS130 GLN132 ASN133 LEU135 ALA145 ASP146 ALA150 ARG151 ALA152 PHE153 GLY154 TYR160 GLU163 VAL164 THR166 LEU167 ARG170 ASP207 SER208
DTL-AVOTACICLIB	− 7.1	502	36, − 8, 15	21 × 21 × 21	GLU 25, ASN 24, MET 40, SER 39, GLN38, LEU 126, LEU 22, ASP 41, GLN 125, VAL 123, ILE 23, SER 42	CDK1-AVOTACICLIB	− 7.6	12,925	− 15, 29, − 34	35 × 35 × 35	LEU135, ALA145, ASP146, ALA150, ARG151, GLY154, HIS162, GLU163, THR166, TYR169, ARG170, SER171, VAL174, LEU175, LEU176, GLY177, SER178, ALA179, TYR181, VAL185, SER189, GLU209, GLN235, ASP236, GLU12, GLY13, THR14, TYR15, VAL18, LYS33, THR47, GLU51, VAL64, LYS89, ARG127, ASP128, GLN132, ASN133.
DTL-ALVOCIDIB	− 7.3	502	36, − 8, 15	21 × 21 × 21	ASP41, MET 40, SER39, ASP 122, GLU 25, SER 42, HIS 44	CDK1-ALVOCIDIB	− 9	6034	− 24, − 8, − 11	35 × 33 × 21	TYR8, SER9, ASP10, LYS11, GLU18, ARG20, ARG44, GLN49, GLN50, SER51, ILE10, GLY11, GLU12, GLY13, VAL18, ALA31, LYS33, PHE80, PHE82, LEU83, SER84, MET85, ASP86, LEU87, LYS88, LYS89, LEU91, ASP92, ILE94, PRO96, PRO131, GLN132, ASN133, LEU135, ASP146, VAL165, TRP168, TYR169, GLU196, LYS200, LYS201, PRO202, HIS205.
DTL-ALSTERPAULLONE	− 7.6	502	36, − 8, 15	21 × 21 × 21	SER 39, ILE 61, VAL75, PHE 91, LEU 54, HIS 96, VAL 93, HIS 73, ILE 99,	CDK1-ALSTERPAULLONE	− 8.7	6689	− 15, − 5, − 28	21 × 35 × 34	ARG44, GLN49, GLN50, ILE10, GLY11, GLU12, GLY13, THR14, VAL18, ALA31, LYS33, VAL64, PHE80, GLU81, PHE82, LEU83, SER84, ASP86, LYS89, LYS130, GLN132, ASN133, LEU135, ALA145, ASP146, TYR160, GLU163, VAL164, LEU167, SER208, GLU209.
DTL-HYMENIADISINE	− 7.1	687	59, 3, − 26	19 × 19 × 19	ILE23, ALA26, VAL 123, GLU 124, CYS 710, SER 42, HIS 44, GLN 38, LEU 47	CDK1-HYMENIADISINE	− 8.4	6120	− 5, 34, − 16	35 × 33 × 19	GLU41, VAL44, ILE10, GLY11, GLY13, THR14, VAL18, ALA31, LYS33, THR47, ALA48, GLU51, VAL64, PHE80, GLU81, PHE82, LEU83, SER84, MET85, ASP86, LYS88, LYS89, ASP92, ARG127, LYS130, GLN132, ASN133, LEU135, ALA145, ASP146, ALA150, ARG151, ALA152, PHE153, VAL165, TRP168, TYR181, TYR7, TYR8, ARG44, GLN49, GLN50.
DTL-OLOMOUCINE	− 6.9	610	48, 35, − 21	22 × 22 × 22	VAL 195, LYS 110, PRO 140, THR 185, ALA 194, SER 186, GLU 193, ASN 107, GLU 143, LEU 182, GLU 109,	CDK1-OLOMOUCINE	− 7.5	6120	− 5, 34, − 16	22 × 35 × 33	ASP27, LYS30, ILE10, GLY11, GLU12, GLY13, THR14, VAL18, ALA31, LYS33, THR47, ALA48, GLU51, VAL64, PHE80, GLU81, PHE82, LEU83, SER84, MET85, ASP86, LYS88, LYS89, ARG127, LYS130, GLN132, ASN133, LEU135, ALA145, ASP146, THR161, HIS162, GLU163, VAL164, VAL165, THR166, TRP168, ARG170, VAL185, GLU209, GLN5, ILE6, TYR7, TYR8, ARG20, PRO25, ILE28, ARG44, LEU46, GLY47, VAL48, GLN49, GLN50, SER51.
DTL-AT7519	− 7.2	502	36, − 8, 15	22 × 22 × 22	MET 40, GLN38, ILE 126, SER 39, CYS 710, HIS44, GLU 25, LEU 121, GLU 124	CDK1-AT7519	− 8	6034	− 24, − 8, − 11	35 × 33 × 22	ARG44, GLN49, GLN50, ILE10, GLU12, GLY13, THR14, VAL18, ALA31, LYS33, LEU37, PHE82, LEU83, SER84, MET85, ASP86, LYS88, LYS89, ARG127, LYS130, GLN132, ASN133, LEU135, ASP146, ARG151, ALA152, PHE153, GLY154, TYR160, HIS162, GLU163, VAL164, THR166, LEU167, ARG170.
DTL-INDIRUBIN-3’-MONOXIME	− 7.2	502	36, − 8, 15	20 × 20 × 20	MET 40, SER 39, ILE 23, LEU 22, ASP 41, GLU 25, SER 42,	CDK1-INDIRUBIN-3’-MONOXIME	− 8.1	6120	− 5, 34, − 16	35 × 33 × 20	GLN49, ILE10, GLY11, GLU12, GLY13, VAL18, ALA31, LYS33, GLU51, VAL64, PHE80, GLU81, PHE82, LEU83, SER84, MET85, ASP86, LYS89, ARG127, GLN132, ASN133, LEU135, ALA145, ASP146, ARG151, ALA152, PHE153.

The 2D schematic diagram revealed that Fisetin formed a complex network of molecular interactions with critical amino acid residues, notably including glutamic acid (GLU, A-143), asparagine (ASN, E-107), and aspartic acid (ASP, E-86). These interactions are characterized by the presence of conventional hydrogen bonds, electrostatic attractions, and van der Waals forces, collectively contributing to heightened binding affinity. Additionally, certain unfavorable acceptor–acceptor interactions may indicate potential steric clashes. The presence of pi-anion and pi-alkyl interactions underscores the intricate binding mechanism of Fisetin with DTL, suggesting its relevance for therapeutic applications based on its competitive binding characteristics.

### Cell viability assay

**Fig. 11:**
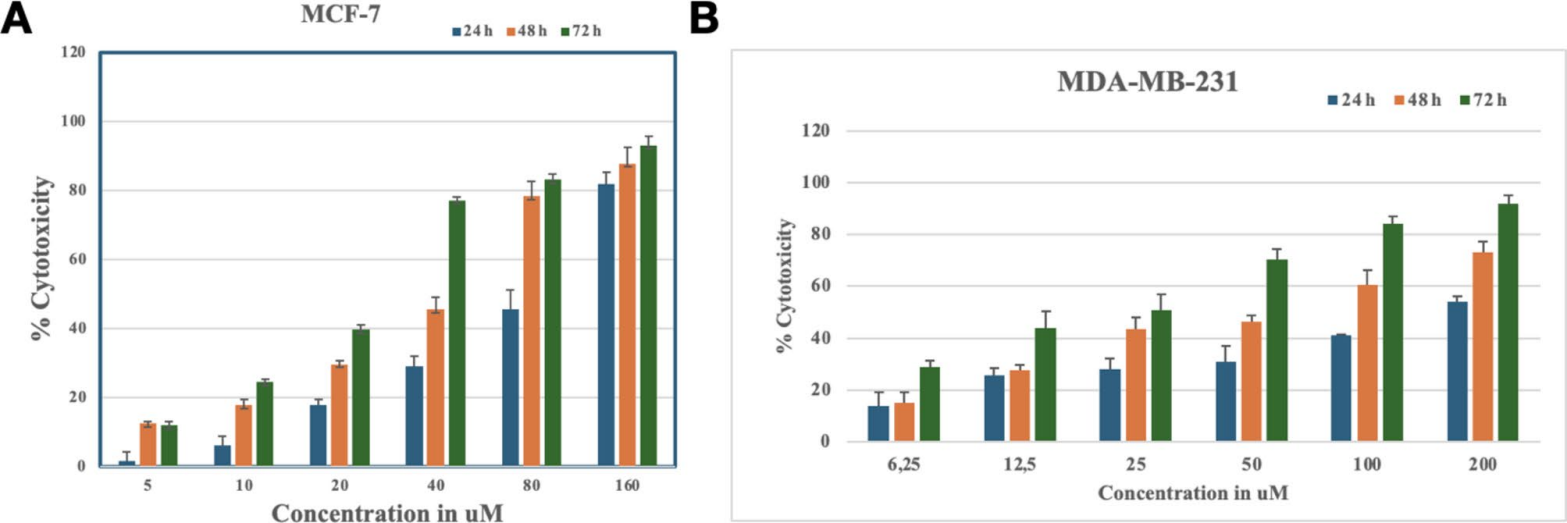
In vitro validation of small molecule drugs. MTT cell viability analysis of (**A**) MCF-7 and (**B**) MDA-MB-231 cells treated with and various concentration Fisetin for three different time points (24 h, 48 h, and 72 h).

Finally, by using cell viability assay, we evaluated the effects of Fisetin in MDA-MB-231 and in MCF-7 (Fig. 11). Fisetin inhibits the viability of MCF-7 and MDA-MB-231 cells. MTT assays were used to investigate the effect of Fisetin on MCF-7 cells. Figure 11 demonstrates the dose-dependent inhibition of MCF-7 and MDA-MB-231 cell viability by Fisetin. Fisetin treatment of 40µmol/L for 24–48 h increased cell toxicity by 45–30% compared with control. Specifically, it was found that the half-maximal inhibitory concentration (IC50) values of Fisetin on MCF-7 cells at 24 h was found to be 88.6283µM. And after 48 h and 72 h, the IC50 values of Fisetin were 40.399µM and 22.780µM respectively. The IC50 values of Fisetin with MDA-MB-231 were 172.129µM, 72.161µM, and 21.746µM for 24 h, 48 h, and 72 h, respectively.

## Discussion

This study aims to explore the complex process of the transition from ductal carcinoma in situ (DCIS) to invasive ductal carcinoma (IDC) in breast cancer. This transition is a critical stage marked by patient and intra-tumoral heterogeneity. While DCIS is considered an early-stage condition, its potential to evolve into invasive cancer is not well understood due to the multifaceted nature of both patient and intra-tumoral differences, making it challenging to fully grasp the underlying mechanisms.

To address this puzzle, our research focuses on unraveling the intricate mechanisms that drive this transformation, specifically honing in on identifying key genes in fibroblasts that play a crucial role in the shift from DCIS to IDC. Using transcriptome sequencing data, we aim to uncover the complexities associated with this pivotal shift in breast cancer development. Our goal is to provide insights that can contribute to a better understanding of the transition process and potentially inform future therapeutic strategies.

In our detailed exploration of the complex molecular landscape, we successfully identified ten specific genes that play a crucial role in fibroblasts during the transition from DCIS to IDC. These genes are CDK1, KIF11, NUF2, ASPM, CDCA8, CENPF, DTL, EXO1, KIF2C, and ZWINT. Importantly, these genes showed strong positive correlations with essential pathways like the cell cycle and DNA repair, highlighting their central roles in guiding the dynamic process of cellular transformation.

To strengthen our findings, we referred to existing scientific literature, finding consistent evidence that supported our identified genes’ connections to fundamental biological processes such as cell cycle regulation, base excision repair, DNA replication, and fatty acid metabolism.

Our analysis went further into exploring protein-protein interactions (PPI) networks using the STRING database. This tool allowed us to gain valuable insights into the functional relationships and interactions among the identified hub genes within the co-expression module. By integrating the STRING database with Cytoscape software, we were able to visualize the interactions among the 150 differentially expressed genes from the module, providing a clear representation of potential molecular mechanisms at play during the transition from DCIS to IDC.

In our exploration of the protein-protein interaction (PPI) network, we identified two distinct clusters with specific biological significance. Cluster 1, with 77 nodes and 458 edges, was linked to processes like Microtubule cytoskeleton organization, Nuclear division, and Nucleobase-containing compound metabolic processes. On the other hand, Cluster 2, consisting of 31 nodes and 13 edges, was associated with functions such as DNA replication, Chromatin remodeling, DNA repair, and Nucleic acid metabolic processes. These enrichments provided valuable insights into the intricate regulatory mechanisms and biological pathways involving the identified hub genes.

To identify these hub genes, we used three different methods: Degree, Maximum Neighborhood Component (MNC), and Maximal Clique Centrality (MCC). The consistent results across all three approaches highlighted ten key hub genes: ZWINT, DTL, EXO1, KIF11, CENPF, ASPM, KIF2C, NUF2, CDCA8, and CDK1. Visualization using Cytoscape emphasized the central roles of CDK1 and DTL in mediating interactions within the network.

Our exploration extended to functional enrichment analysis, using Gene Ontology (GO) and pathway analyses to gain deeper insights. The GO annotation revealed significant involvement in processes such as sister chromatid segregation, nuclear division, chromosome segregation, spindle organization, and terms related to cellular components and molecular functions. KEGG pathway analysis highlighted connections with pathways including the cell cycle, DNA replication, and nucleocytoplasmic transport.

We went even deeper into regulatory networks by identifying transcription factors (TFs) and miRNAs associated with the hub genes. The identification of 43 prominent TFs within the brown module showcased the diverse range of regulatory mechanisms. Additionally, 22 miRNAs were identified as regulators of target genes, with miR-193b-3p displaying significant regulatory potential.

To validate the clinical significance of the identified hub genes, we conducted survival analysis, revealing a correlation between higher expression of these genes and poorer patient survival. ROC curve analysis further provided evidence of the high predictive accuracy of ten hub genes for the development of invasive ductal carcinoma (IDC) after ductal carcinoma in situ (DCIS).

Elevated CDK1 expression is a common feature in over 20 human tumors, making it a potential immunotherapy target^23^. In breast cancer, CDK1, CDK5, and CDK20 are overexpressed, while CDK2 and CDK6 are decreased, with high expression correlating with poor prognosis^24,25^. Dysregulation of CDKs, especially CDK1, contributes to increased cell proliferation in breast cancer, suggesting selective CDK1 inhibition as an effective treatment strategy^23^. It is proposed that CDK1 would be the best CDK target for breast cancer therapy since selective blockade of CDK1 alone or in combination with other therapies has been linked to strong anti-cancer results^25,26^. Serine/threonine kinases known as cyclin-dependent kinases (CDKs) have catalytic activity that are controlled by their interactions with cyclins and CDK inhibitors (CKIs). CDKs are essential regulatory enzymes that control transcriptional processes and cell-cycle checkpoints in response to external and intracellular cues, hence promoting cell proliferation. It should come as no surprise that dysregulation of CDKs is a common feature of malignancies, and that inhibiting particular members of the protein is a desirable target for cancer treatment. The Food and Drug Administration (FDA) recently approved dual CDK4/6 inhibitors, palbociclib, ribociclib, and abemaciclib in combination with other agents for the treatment of hormone receptor positive (HR+) advanced or metastatic breast cancer (A/MBC), as well as other subtypes of breast cancer. Moreover, more specific CDK inhibitors have been found to be viable therapeutic targets by continuing research^27–31^.

It has previously been demonstrated that greater miRNA expression rates are expected to happen after miR-mimic transfection. However, in the setting of several cell-lines, the observed divergent patterns of miRNA grow, suggesting the participation of increasingly intricate cellular processes. Previous research comparing aggressive MDA-MB-231, MDA-MB-453, and BT549 breast cancer cell lines to normal mammary epithelial cells revealed over-expression of DDAH1. There found an adverse correlation between the microRNA miR-193b and DDAH1 expression. Ectopic expression of miR193b decreased the conversion of ADMA to citrulline and DDAH1 expression in DDAH1 + MDA-MB-231 cells. When miR-193b was inhibited, DDAH1 expression was increased in DDAH1 − MCF7 cells. Assays using luciferase reporter showed that miR-193b directly targets DDAH1. An in vitro study of VM using MDA-MB-231 cells organised into tube structures was significantly suppressed by either miR-193b expression or DDAH1 silencing. Mechanistically, they discovered that DDAH1 knockdown impeded cell migration whereas miR-193b controlled MDA-MB-231 cells’ migration and proliferation. These results demonstrate that targeting DDAH1 expression and/or enzymatic activity may be a viable strategy in the treatment of aggressive breast malignancies. They also constitute the first evidence for DDAH1 expression, regulation, and function in breast cancer cells^15–18,32^.

Another significant gene, DTL, part of the ubiquitin-proteasome system involving CUL4A, shows elevated expression in cancers, and DTL knockdown hinders cancer cell functions. PDCD4, a tumor suppressor, regulates cancer progression^33^. In invasive breast cancer, DTL is significantly upregulated, with lower expression correlating with better overall survival in luminal subtypes^34,35^. In cervical adenocarcinoma, overexpressed DTL is associated with a poor prognosis, promoting cell migration and invasion via the RAC1-JNK-FOXO1 axis^23^. Osteopontin induces DTL expression in liver cancer cells through PI3K/AKT signaling, promoting cancer cell growth and invasion. DTL/RAMP, significantly upregulated in breast cancer, exhibits cell-cycle-dependent localization and highest expression at G1/S phases. Its phosphorylation by Aurora kinase-B affects stability, and depleting DTL/RAMP inhibits breast cancer cell growth, proposing it as a potential therapeutic target^36^.

Proteins involved in ubiquitination and degradation, such as UBE2T and DTL in breast and lung tumors, are associated with poor outcomes, suggesting their potential as therapeutic targets^37^. Fisetin, a flavonoid compound, shows therapeutic potential by impacting inflammation, oxidative stress, and metabolic pathways^37^.

Hence, targeting CDK1 and DTL presents promising avenues for cancer therapy, particularly in breast cancer, emphasizing the need for further research and clinical trials to establish their efficacy. Our study highlights Fisetin’s potential benefits but stresses the importance of rigorous clinical trials for conclusive evidence. Previous studies support Fisetin’s pharmacological attributes and underscore the need for more trials. Our findings contribute to understanding Fisetin’s potential interventions in various health conditions, emphasizing the need for further research. In conclusion, our study combines bioinformatics analyses and in vitro analyses to unravel complex molecular mechanisms, shedding light on disease progression and treatment strategies. Future directions should include robust molecular investigations alongside comprehensive in vivo and in vitro assays to explore transformative processes and implications for disease progression and treatment strategies^38,39^.

In terms of previous studies, there are a number of studies which have been carried close to the similar work but not in breast cancer^11,12,40–50^. There are also some works which show the simplified approach for predicting the target proteins in other cancer types such as ovarian cancer and other human diseases based on high-throughput data and use herbal drugs to target the genes/proteins^6,38,39,51–58^. In this work, WGCNA was applied for predicting hub genes and putative biomarkers in different human diseases mainly cancers. Similar to it, we carried out the work which predicts the hub genes and also targeting target proteins inferred from the hub genes and finally, we performed cell viability assay. The limitation of the study is that we have used the previously published datasets and the positive perspective is that it could be easily implemented for in-vitro and in-vivo samples.

## Materials and methods

### Data collection

From the Genome Expression Omnibus (GEO), we downloaded fibroblast-derived pre-invasive and invasive ductal carcinoma mRNA expression datasets. In this study, GSE228582 (url: https://www.ncbi.nlm.nih.gov/geo/query/acc.cgi?acc=GSE228582) was utilized to build a weighted gene coexpression network using the WGCNA R-package. GSE228582 included 6 DCIS patients, 6 IDC patients, and four normal tissues. The platform used for gene expression profiling was GPL16791 Illumina HiSeq 2500 (*Homo sapiens*). Top 10 hub genes have been found in DCIS/IDC patients. The Genome Expression Omnibus is a public archive of RNA-seq, microarray, chip, and other high throughput sequencing data hosted by Nation Centre for Biotechnology Information^59^. A user can download data from the database and then analyze it using its web-based tools. We used openly accessible datasets in the present study^51,52,55,57,60^.

### Prediction of differentially expressed genes

Raw counts of the datasets were downloaded and used to measure transcriptomic gene expression levels. The data was normalized by RMA normalization on probe level information to achieve a uniform sample distribution, assuming that all the samples are obtained from a single parent class^61^. Differentially expressed genes (DEGs) are genes that exhibit significant changes in their expression levels between different conditions or groups. DEGs offer insights into biological processes, disease mechanisms, and drug responses^62,63^. There are various techniques available for measuring gene expression, including microarray analysis and RNA sequencing (RNA-seq). Microarray analysis involves simultaneously measuring the expression levels of thousands of genes using microarray chips^9^. The evaluation of DEGs includes hybridizing RNA to gene-specific probes on microarray chips or converting RNA to cDNA, sequencing it, and mapping to a reference genome/transcriptome in RNA-seq^64^. Statistical analysis identifies DEGs based on read counts, considering factors such as sample size and experimental design. Validation is often performed using independent methods like quantitative real-time polymerase chain reaction (qRT-PCR).

### Construction of weighted gene co-expression network

For Weighted Gene Co-Expression Network Analysis (WGCNA), the block-wise approach was employed to construct separate co-expression networks for distinct sample groups or conditions^10,12,65^. This strategy enables the identification of condition-specific modules and the detection of relationships specific to each group. The block-wise WGCNA process entails data preprocessing, correlation calculation, construction of an adjacency matrix, thresholding, calculation of the Topological Overlap Measure (TOM), hierarchical clustering, and determination of module eigengenes and module membership^66^. To initiate the analysis, an expression matrix was created using Differentially Expressed Genes (DEGs), with columns representing samples or conditions and rows representing genes. Rigorous quality checks were conducted to identify and eliminate outliers or low-quality samples, ensuring the robustness of subsequent network analysis^67^. Subsequently, pairwise correlations between genes were computed utilizing an appropriate correlation metric, such as the Pearson correlation coefficient^68^. The Pearson correlation coefficient between genes *i* and *j* is calculated using the formula:

$$\:cor(i,j)=\frac{cov({x}_{i},{x}_{j})}{\left(sd\left({x}_{i}\right)*sd\right({x}_{j}\left)\right)},$$

where $\:{x}_{i}$ and $\:{x}_{j}$ represent the expressions of genes i and j, respectively.

The absolute value of the Pearson correlation coefficient is used to determine the similarity between genes. These similarity values are then transformed into adjacency values using a power function^69^. The formula for soft-thresholding is:

$$\:{a}_{ij}={\left|cor\right(i,j\left)\right|}^{\beta\:}.$$

Here, $\:cor(i,j)$ is the Pearson correlation coefficient between genes i and j, and β is the soft-thresholding power that determines the scale-free topology of the network.

The separate adjacency matrices obtained from each group were combined into a composite adjacency matrix, representing the overall network structure across all sample groups. A threshold was applied to composite adjacency matrix to convert it into a binary adjacency matrix. The threshold is determined by selecting the soft-thresholding power β that yields the highest scale-free topology fit index. The Topological Overlap Measure (TOM) was then calculated based on the concept of shared neighbors, considering both direct and indirect connections between genes. The TOM value between genes *i* and *j* is computed using the formula:

$$\:TOM(i,j)=\frac{(a+b)}{({min}\left({k}_{i},{k}_{j}\right)+1-(a+b\left)\right)},$$

where a represents the number of shared neighbors between genes *i* and *j*, b represents the number of neighbors exclusive to either gene *i* or gene *j*, and $\:{k}_{i}$ and $\:{k}_{j}$ represent the total number of neighbors for genes i and j, respectively. TOM values range between 0 and 1, with higher values indicating stronger connectivity or similarity between genes.

Then, hierarchical clustering was performed on the TOM-based dissimilarity matrix to group the genes into different modules. The TOM-based similarity between genes *i* and *j* is calculated using the formula:

$$\:TOM\left(i,j\right)=1-dissimilarity(i,j).$$

The dissimilarity matrix is derived from the Topological Overlap Measure (TOM) values and serves to illustrate the pairwise distances or dissimilarities between genes within the network. Hierarchical clustering algorithms, such as average linkage or complete linkage, can be applied to this dissimilarity matrix to construct dendrograms, which visually depict the clustering of genes^70^. In the subsequent steps, we computed the module eigengene and module membership. The module eigengene condenses the collective expression pattern of genes within a module into a single representative profile. Meanwhile, the concept of module membership in WGCNA refers to the extent of correlation or association between each gene and a specific module within a gene co-expression network^10^. This metric quantifies how well a gene aligns with a particular module based on its expression pattern.

The formulas for calculating module eigengene and module membership are as follows:

$$\:Eigengene\left(i\right)=\frac{\sum\:\left(jinmodule\right)({a}_{ij}\times\:{x}_{j})}{\sum\:\left(jinmodule\right)\left({a}_{ij}\right)},$$

where $\:{a}_{ij}$ represents the adjacency value between genes i and j, and $\:{x}_{j}$ represents the expression of gene j within the module.

$$\:Modulemembership\left(i\right)=cor({x}_{i},Eigengene(module\left)\right),$$

where $\:{x}_{j}$ represents the expression of gene i, and Eigengene (module) represents the eigengene of the module

### Selection of key modules based on clinical traits

Key modules were selected based on clinical traits using WGCNA. The module eigengenes were correlated with the clinical traits of interest. This was done by calculating the correlation coefficient between the module eigengene values and the corresponding clinical trait values. Positive correlations indicate a positive association between the module and the clinical trait, while negative correlations indicate a negative association. Statistical tests determined significant module-trait associations, Commonly used tests include linear regression analysis or permutation tests^12^. Key modules were selected based on their module significance and gene significance. Modules with high module significance and genes with high GS values are considered important for the clinical traits of interest. These key modules represent gene expression patterns that are strongly associated with the clinical phenotype^71^.

### Functional enrichment analysis of specific modules of interest

In our study, we conducted functional enrichment analysis of specific modules to gain a deeper understanding of the biological functions, processes, and pathways associated with the genes within those modules. This analysis was facilitated through gene ontology (GO) analysis, which categorizes genes based on their involvement in various biological processes, molecular functions, and cellular components. To carry out this analysis, we employed the iDEP (integrated Differential Expression and Pathway analysis) web-based tool. iDEP offers a comprehensive suite of functional enrichment analysis tools, including GO analysis and pathway analysis, enabling us to delve into the functional implications of the genes within our modules of interest^72^.

The iDEP platform, accessible at http://bioinformatics.sdstate.edu/idep/, proved invaluable in providing insights into the biological context of the examined genes. iDEP incorporates the hierarchical structure of Gene Ontology (GO) terms, which allows for the identification of enriched terms at various levels, thus providing a comprehensive understanding of biological processes, molecular functions, and cellular components. Additionally, iDEP utilizes pathway databases such as the Kyoto Encyclopedia of Genes and Genomes (KEGG) and Reactome to detect enriched pathways^73–75^. This analysis assesses the statistical significance and enrichment of genes within each pathway, aiding in the elucidation of their potential roles^74^. To determine the statistical significance of enrichment results and account for multiple testing, iDEP employs methods such as the Benjamini-Hochberg correction for false discovery rate (FDR). This correction helps ensure that significant associations are not due to random chance. Furthermore, iDEP calculates the fold enrichment, a measure of how much more prevalent a particular category of genes is within a module compared to the background. A higher fold enrichment value indicates a stronger association of genes within a specific category^76^.

### Identification of transcription factors (TFs) within the key module

The identification of transcription factors (TFs) within the key modules was performed by Network Analyst tool. Network Analyst offers a range of features tailored to streamline gene expression analysis and interpretation. Leveraging diverse data sources, such as ENCODE, JASPAR, and ChEA, researchers can seamlessly interrogate transcriptional regulatory networks, pinpoint potential transcription factors (TFs), and decipher their roles in orchestrating gene expression patterns. The platform’s intuitive interface allows users to interactively visualize, analyze, and compare data from different sources, enabling the exploration of complex regulatory relationships within biological systems.

### Identification of miRNA in the key module

We used miRTarBase for miRNA Identification which is a widely used web-based bioinformatics tool that enables researchers to analyze gene lists in relation to biological pathways, gene ontology terms, transcription factor binding sites, miRNA target predictions, and other gene set libraries^77^. It facilitates functional enrichment analysis and helps researchers understand the biological implications of their gene lists. By utilizing miRNA target databases such as miRTarBase and TargetScan, Enrichr identifies miRNAs that potentially target the genes within a given module. The analysis algorithm incorporates this information to predict miRNA-gene interactions, providing valuable insights into the regulatory relationships within the module and expanding our knowledge of miRNA involvement in the studied context^11,78^.

### Identification of hub genes

Maximum Neighborhood Component (MNC) algorithm to rank the nodes (genes) based on their significance as common hub genes within the PPI network.

To identify hub genes within selected modules, we initially calculated module membership (MM) values for each gene. These MM values reflect the correlation between gene expression and the module eigengene, indicating the gene’s association with the overall expression pattern of the module. Genes exhibiting high MM values, indicative of a strong correlation, were identified as potential hub gene candidates within their respective modules^12^.

Subsequently, the STRING web tool (https://string-db.org/) was employed to explore potential interactions among the identified hub genes. STRING offers valuable insights into protein-protein interactions (PPI) and assigns confidence scores to these interactions. To ensure reliability, we implemented a confidence score threshold of 0.4 for filtering PPI interactions. This threshold ensured the inclusion of interactions with equal to or higher confidence levels, enhancing the reliability and meaningfulness of the interactions selected^79^.

Upon exporting the high-confidence PPI network data, along with gene nodes and their associated confidence scores, from STRING, we imported this data into Cytoscape. Within Cytoscape, we harnessed the cytoHubba plugin, which offers a range of algorithms designed for the identification of hub genes based on diverse topological measures. For our specific analysis, we applied the MNC algorithm to rank the nodes (genes) according to their significance as common hub genes within the PPI network^13,53,57,80,81^.

### Docking analysis

In our study, we conducted molecular docking simulations to investigate the effects of CDK1 inhibitors on the DTL hub gene within the context of Invasive Ductal Carcinoma. The molecular structures of CDK1 and DTL proteins were obtained from the Protein Data Bank (PDB) database (https://www1.rcsb.org/). Subsequently, water molecules and pro-ligands were removed from the target structures using PyMOL 2.3.0. The CDK1 inhibitors, including Fisetin, Doxurubicin, Alsterpaullone, Alvocidib, Hymenialdisine, Indirubin-3’-monoxime, Olomoucine, AT-7519, Fostamatinib, and Avotaciclib, were sourced from the PubChem database (https://pubchem.ncbi.nlm.nih.gov/). The target protein structures underwent preparation steps using Discovery Biovia Studio (Biovia, Dassault Systèmes, 2021), which involved hydrogenation and optimization of the conformation of CDK1 inhibitors as well as determination of torsional bonds^54,55,82,83^.

To predict the active pockets of the proteins, we utilized the POCASA tool. The docking range was established within the predicted active pocket, and the pertinent docking range information was retained for subsequent docking procedures. Molecular docking simulations between the target proteins and CDK1 inhibitors were carried out using AutoDock Vina v.1.2.0^82^.

### MTT assay

MCF-7 cell line was obtained from the National Centre for Cell Sciences (NCCS) in Pune, India. Cells were grown as monolayer cultures in Dulbecco’s modified Eagle’s medium (DMEM) containing 10% fetal bovine serum (FBS), 1% penicillin-streptomycin (50 mg/ml in a humidified atmosphere of 5% CO_2_ at 37 °C in T-25 culture flasks were sub-cultured twice a week.

After incubation, the medium was removed, and the cells were cultured in fresh growth medium containing 0.5 mg/mL MTT for 4 h under the same conditions. Subsequently, the supernatant was removed, and 100 µL DMSO was added to dissolve the formazan crystals. The optical density (OD) value was measured at 570 nm using a microplate reader (Thermo Fisher Scientific, USA). The results were expressed as the half-maximal inhibitory concentration (IC50), representing the concentration of the chemotherapeutic drug required for 50% inhibition in vitro.

To assess cell viability, the MTT assay was utilized. In 96-well culture plates, the cells were planted at a density of 1 × 10^4^ cells/well. Different Fisetin concentrations (5, 10, 20, 40, 80, 160 µmol/L) were added after 4 h, and the mixture was then incubated for 24 h, 48 h and 72 h. As a control, cells were treated in culture media containing a similar volume of dimethyl sulfoxide (DMSO). To calculate the cell survival rate, the absorbance was measured using a microplate reader at 570 nm excitation/emission wavelengths.

## Conclusions

In conclusion, our research underscores the intricate molecular landscape of DCIS and IDC progression. The integrated analysis of hub genes, PPI networks, functional enrichments, and regulatory factors provides comprehensive insights into the underlying mechanisms. CDK1 and DTL emerged as central players, suggesting their critical roles in the regulatory pathways. These findings hold promise for advancing our understanding of breast cancer progression and potential therapeutic interventions.

## Data Availability

The datasets generated and analyzed during the current study are available in the given references in the freely available public database followed by the accession number. We have also provided the accession numbers for the datasets. All resources have been provided and are accessible.
